# Socio-Economic Differences in the Prevalence of Single Motherhood in North America and Europe

**DOI:** 10.1007/s10680-021-09591-3

**Published:** 2021-07-13

**Authors:** Judith C. Koops, Aart C. Liefbroer, Anne H. Gauthier

**Affiliations:** 1 Netherlands Interdisciplinary Demographic Institute (NIDI)-KNAW/University of Groningen, The Hague, The Netherlands; 2 Department of Epidemiology, University Medical Centre Groningen, Groningen, The Netherlands; 3 Department of Sociology, Vrije Universiteit Amsterdam, Amsterdam, The Netherlands; 4 Faculty of Behavioural and Social Sciences, University of Groningen, Groningen, The Netherlands

**Keywords:** Childbearing, Marriage, Single parenthood, Europe, North America, Fertility

## Abstract

The study focuses on understanding the association between parental socio-economic status (SES) and the likelihood of women experiencing a first birth while single, and identifying societal factors that influence this association in 18 North American and European societies. Previous research has shown that single motherhood occurs disproportionately among those from with lower a lower parental SES. The study assesses whether this is caused by parental SES differences in the risk of single women experiencing a first conception leading to a live birth or by parental SES differences in how likely women are to enter a union during pregnancy. Additionally, an assessment is made of whether cross-national differences in these associations can be explained by a country’s access to family planning, norms regarding family formation, and economic inequality. Across countries, a negative gradient of parental SES was found on the likelihood of single women to experience a first pregnancy. The negative gradient was stronger in countries with better access to family planning. In some countries, the negative gradient of parental SES was aggravated during pregnancy because women from lower parental SES were less likely to enter a union. This was mostly found in societies with less conservative norms regarding marriage. The results suggest that certain developments in Western societies may increase socio-economic differentials in family demography.

**Supplementary Information:**

The online version contains supplementary material available at 10.1007/s10680-021-09591-3.

During the first half of the twentieth century, strong social norms dictated that sexual intercourse, childbearing, and child rearing should take place within marriage. This period was characterized by high marriage rates, low divorce rates, and ‘shotgun’ marriages in reaction to accidental pregnancies (Axinn and Thornton 2000; Sobotka and Toulemon 2008). Since then, many Western societies have displayed a greater acceptance of sex and building a family outside of marriage (Sobotka and Toulemon 2008). The disconnection between marriage and childbearing is reflected in an increase in cohabiting parents and single parenthood (Ellwood and Jencks 2004; Härkönen 2016; Heuveline et al. 2003; Kiernan 2004; Lichter et al. 2014; Perelli-Harris et al. 2012). These changes resulted from different—but often interrelated—forces at the societal level, such as the rise of individualistic values emphasizing self-expression and self-fulfilment, the decreasing importance of religious institutions, and the technical advancement of contraceptive methods (Lappegård et al. 2018; Lesthaeghe 2010).

Not everyone is equally likely to become an unmarried parent. Non-marital parenthood occurs disproportionately among those from lower socio-economic backgrounds (Aassve 2003; Amato et al. 2008; Koops et al. 2017; Mikolai et al. 2018; Perelli-Harris, Sigle-Rushton, et al. 2010a, b). This is especially the case for parents who do not live together with a partner, henceforth referred to as single parents (Härkönen 2016; Musick 2002). Moreover, compared with two-parent families, poverty rates are higher among single-parent families (Brady and Burroway 2012; Kollmeyer 2013; McLanahan 2009). This inequality is even observed in societies with generous social policies that are either universal or targeted towards countering poverty among single-parent families (Brady and Burroway 2012). This has led to concerns of ‘diverging destinies’, as a result of socio-economic differentiation in family demography, among American (McLanahan 2004) and European scholars (Härkönen 2016; Kollmeyer 2013).

The aim of the current study is to deepen our understanding of the association between socio-economic background and single motherhood across a large number of Western societies. To clarify, in this study we use the term ‘single’ (e.g. single women, single motherhood), to refer to a situation where a person does not live together with a partner. Socio-economic background is captured with parental socio-economic status (SES) rather than women’s own SES. Thus far, cross-national research has mostly examined the influence of achievement (socio-economic success based on personal talents and skills) on single motherhood by focusing on own SES (Mikolai et al. 2018; Perelli-Harris, Sigle-Rushton, et al. 2010a, b). However, the literature suggests that ascription (socio-economic success on the basis of birth) remains to play an important role in women’s likelihood of experiencing a first birth while single (Aassve 2003; Amato et al. 2008). According to this literature, parental SES can influence single motherhood through own educational achievement, for example through the mechanism of school engagement and school dropout (Harden et al. 2009; Imamura et al. 2007). In addition, women with lower parental SES may receive less monitoring and supervision (Hofferth and Goldscheider 2010), feel less in control over their lives (Musick et al. 2009), have fewer educational and occupational aspirations (Frisco 2005), and may receive less material and financial support (Albertini and Kohli 2013). A methodological advantage of using parental SES is that it does not suffer from reverse causality with single motherhood, whereas a woman’s own SES often does (Hoem and Kreyenfeld 2006).

First, we will examine at which stage during family formation differences in single motherhood come about. Is it caused by parental SES differences in the risk of single women to become pregnant, parental SES differences in the risk of single women who are pregnant to start living as a couple before childbearing, or both? Second, we will examine whether the association between parental SES and single motherhood differs across societies. Can cross-national variation in the association of parental SES with single motherhood perhaps be explained by societal factors related to access to family planning, norms regarding family formation, and socio-economic conditions? We will briefly outline the contributions of this study to the literature.

Single mothers are often regarded as a single group, covering women who did not live with a partner upon entry into motherhood, those who separated from their partner after they became a parent, and those who lost their partner through death. In the current study, we focus on the first of these groups. Research has shown that economic hardship—already more prevalent among single mother families than among two-parent families—is even higher among never-married single mothers (Korenman et al. 2001). Among the reasons for this are that fathers are less likely to be involved or to contribute financially to mothers and children with whom they have never lived (Carlson 2006; Kane et al. 2015). The inherent lack of resources is hard to offset by other means because unemployment rates are high among these single mothers (Härkönen et al. 2016), and many of them—especially those with lower parental SES—remain unpartnered for prolonged periods (Kalmijn and Monden 2010). The first contribution of this study is to focus on the group of single mothers who were single when they entered motherhood and who form an important link in explaining the socio-economic differentiation in family formation.

The second contribution of this paper is an examination of parental SES differences at two subsequent points in the life course, at the moment of conception and during pregnancy. Research has shown that births to single women are more common among those with lower parental SES. However, the stage during family formation at which these differences come about remains unclear. It is possible that women with lower parental SES are more likely to become pregnant outside of a union than women with higher parental SES (Fig. 1, Arrow 1). This would accord with the literature suggesting that women with higher parental SES are more motivated to avoid unplanned pregnancies and are better at preventing it (Miller 2002; Smith et al. 2018). It is also possible that women with lower parental SES are less likely than women with higher parental SES to enter a union once pregnant (Fig. 1, Arrow 2). This may be the result of better access to (financial) resources of women with higher parental SES, which increases their ability to move in with a suitable partner.

**Fig. 1:**
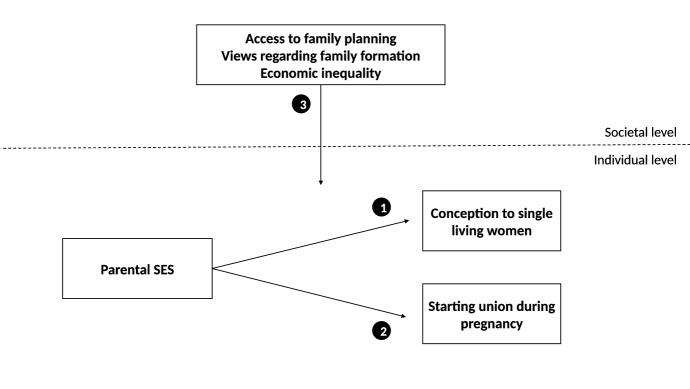
Overview of the conceptual model

In the next step, we look for cross-national differences in the association of parental SES with the likelihood of women experiencing a first birth while single, and assess the extent to which these differences can be explained by macro-indicators (Fig. 1, Arrow 3). Western societies differ in terms of access to family planning, norms regarding family formation, and economic inequality. We argue that these factors may influence socio-economic differences in single motherhood, but at different stages in the process of family formation. Access to family planning may be particularly important to explain socio-economic differences in the risk of single women to become pregnant, whereas norms regarding family formation may be particularly important to explain socio-economic differences in the likelihood of pregnant women to enter a union. Economic inequality may be important at both stages of the family-building process, by influencing the socio-economic situation of women with lower or higher parental SES. By examining these relationships in detail, we contribute to the literature by providing insight into factors that influence parental SES differences at the start of single motherhood and may point to ways in which this inequality can be prevented.

The study uses data from the Generations and Gender Survey, the Harmonized Histories, and the Canadian General Social Survey. These data contain retrospective information on the timing of childbirth and union formation, and allow us to examine transition into single motherhood in 18 Western societies, including North America, and Eastern, Central, Western, and Northern Europe. The data do not contain information on pregnancies that have not been brought to term. Therefore, the data do not allow to examine socio-economic differences in conception rates separately from socio-economic differences in termination of pregnancies through abortion or miscarriage. Rather, we study the accumulation of both processes by examining socio-economic differences in single women’s conception rates leading to a live birth.

## Background

Even though the link between marriage and childbearing has weakened, the norm of two parents taking care of children as the ideal family continues to persist and is widespread throughout Western societies (Stavrova and Fetchenhauer 2015). Having children is costly, both financially and in terms of time. Living together allows parents to pool resources and to specialize in different activities, which makes it a more efficient arrangement than raising children outside of a union (Becker 1981). In addition, single motherhood can interfere greatly with socio-economic prospects, such as educational attainment, occupational aspirations, and marital prospects (Lichter et al. 2014). Even though single motherhood may not be considered ideal in Western societies, and most women do not actively choose it (Cherlin et al. 2008), roughly one in ten women in North America and Europe live without a partner upon entering motherhood (Koops et al. 2017). We now discuss the literature that explains socio-economic differences in the occurrence of single motherhood. Throughout the remainder of the article, single motherhood refers only to women who are not living with a partner when they become a mother; it does not refer to women who became single mothers later in life through divorce, separation or death of a partner.

### Parental SES Differences in Single Motherhood

Three key mechanisms are mentioned in the literature to explain parental SES differences in the likelihood of becoming a single mother. These refer to the idea that women with higher parental SES (1) are expected to be more motivated to avoid becoming a single mother, (2) are expected to be less likely to experience single motherhood at a young age, and (3) are expected to be more successful in preventing single motherhood than women with lower parental SES. We now discuss these mechanisms in more detail.

Although a majority of women may prefer to live with a partner upon entry into motherhood, the negative consequences of single motherhood are assumed to be greater for women with higher parental SES, because they have higher educational and occupational aspirations than women with lower parental SES (Frisco 2005). In addition, the literature suggests that because women with lower parental SES have fewer chances to advance in other areas of their lives, they rank motherhood higher than other options (Edin and Kefalas 2005; Musick 2002), and may indeed welcome—even unplanned—motherhood in order to reduce uncertainty about their future (Friedman et al. 1994; Musick et al. 2009). Overall, women with higher parental SES are therefore expected to be more motivated to avoid becoming a single mother than women with lower parental SES.

Due to the intergenerational transmission of educational opportunities, women with lower parental SES tend to have lower educational achievements than women with higher parental SES (Breen and Jonsson 2005; Jerrim and Macmillan 2015). This may lead to lower school engagement and more school dropout, which can in turn increase the prevalence of pregnancies and single motherhood among these teenagers (Duncan and Hoffman 1990; Harden et al. 2009; Imamura et al. 2007; Manlove 1998). As such, the intergenerational transmission of educational attainment may in part explain why women with lower parental SES are more likely to experience single motherhood, in particular at a young age.

Women with higher parental SES may not only be more motivated to avoid single parenthood, they might also be more successful in pursuing this. Research shows that unplanned pregnancies are more common among women with lower parental SES (Musick et al. 2009). There are several ways in which women can avoid single motherhood: (1) they can postpone sexual initiation until the moment they are in a committed co-residential relationship, (2) they can avoid a pregnancy by using contraceptives, (3) they can terminate a pregnancy if it is detected sufficiently early, or (4) they can move in with a partner during pregnancy. Of these options, differences in terms of postponement of sexual initiation until union formation is the least likely to contribute to parental SES differences found in single motherhood. Research shows that for decades now, sexual initiation has taken place well before the first cohabiting relationship in North America and Europe, and this applies to all socio-economic groups (Teitler 2002). Parental SES differences in entering a union do exist. In Europe and North America women with lower parental SES enter their union earlier than women with higher parental SES (Brons et al. 2017; Sassler et al. 2018). However, this mechanism cannot explain why unplanned pregnancies are more common among women with lower parental SES, because entering a union sooner should actually reduce the likelihood to experience a conception outside a union.

Differences in contraceptive use are mentioned in the (mostly American) literature as a potential source of the parental SES gradient in single motherhood. Women growing up with lower SES parents are expected to receive less monitoring and supervision than their peers with higher SES parents (Hofferth and Goldscheider 2010), due to more stress in the family home and less quality time with parents (Baizán et al. 2014; Bianchi et al. 2004). As a result, they are less likely to use proper contraception (Miller 2002). In addition, growing up with lower SES parents is believed to have a long-term impact by impairing one’s feeling of efficacy and sense of control over circumstances, resulting in a self-fulfilling prophecy in the reduced use of contraceptives (Musick et al. 2009). This, in combination with their more turbulent lives (England and Edin 2007; Smith et al. 2018) and reduced access to medical care (Silverman et al. 1987), means that women with lower parental SES are less likely to use (effective) contraception, or more likely to use it inconsistently (Miller 2002), leading to a higher risk of single motherhood.

Parental SES differences may also arise due to inequalities in access to family planning methods. Children from higher SES parents are generally in a better financial situation than their peers with lower parental SES. Although contraception can be relatively cheap, the costs of an abortion as well as of certain modern methods can be substantial in some countries and may be another reason why people with lower parental SES might forgo using contraceptives or an abortion (Boussen 2012; Musick et al. 2009). Taking all the above factors into account, we hypothesize that:

#### **H1**

Conceptions leading to a live birth are more common among single women with lower parental SES than among single women with higher parental SES.

A higher parental SES tends to be associated with better access to jobs that are more stable and provide a higher income (Breen and Jonsson 2005). In addition to their own incomes, young adults with higher parental SES are more likely to receive material and financial support from their parents in the form of money or transfers of real estate (Albertini and Kohli 2013). The better financial conditions of women with higher parental SES may increase their ability to move in with a partner within the short period of the pregnancy, for example, because they can afford housing. Women with a higher parental SES may also date more ‘attractive’ partners with better economic prospects, which may increase her motivation and that of her partner to commit to the relationship by moving in together (Aassve 2003; Cherlin 2016). Taking these arguments together, we hypothesize that:

#### **H2**

Single women with lower parental SES are less likely to enter a union during pregnancy than women with higher parental SES.

### Cross-National Variation in the Influence of Parental SES

The foregoing theories have mostly been developed and tested in the American context. Cross-national research in fact shows that the association between parental SES and union status upon the birth of the first child varies across countries (Koops et al. 2017). We identify three societal factors that may explain cross-national differences in this association.

The first factor is access to family planning. American research shows that differences in single motherhood may be related to lower contraceptive use and lower abortion rates among women with lower parental SES. However, access to family planning differs greatly across Western societies (Alkema et al. 2013). This may particularly influence parental SES differences in the risk of conception for single women. However, the direction of this effect is less clear. On the one hand, it could be argued that in countries with better access to family planning, women with higher parental SES are more likely to use this option, due to the more severe consequences of becoming a single parent for this group. If this is true, the association of parental SES with the likelihood of conception for single women is stronger in societies with better access to modern contraceptives and higher abortion rates. Alternatively, one could argue that in societies where access to family planning is limited, women with higher parental SES have the means to access it due to their better material and financial resources, whereas this effect disappears when modern contraceptives are more readily available to the wider public. If this is true, the association between parental SES and the likelihood of conception for single women is weaker in societies with better access to modern contraceptives and higher abortion rates.

The second factor relates to norms regarding family formation. In Western societies, to raise children within the context of marriage might still be perceived by many as the ideal family setting (Stavrova and Fetchenhauer 2015). However, research also shows that this perception is changing. Western European societies have become more accepting of alternative family forms (Axinn and Thornton 2000; Sobotka 2008; Sobotka and Toulemon 2008). This may reduce stigmatization and any perceived negative consequences of single motherhood, and may therefore influence the association between parental SES and the likelihood of entering a union during pregnancy. One could argue that, regardless of parental SES, in more conservative societies women are more eager to avoid becoming a single mother in order to escape social stigma. In societies that are more open to alternative forms of family, becoming a single mother may be less of an issue. This would be especially true for women with lower parental SES, because they may perceive less of a disadvantage in becoming a single mother. In this case, the association of parental SES with the likelihood that one will remain single during pregnancy is stronger in societies that are less conservative in their views on family formation.

The third factor is economic inequality. American research indicates that young women with lower parental SES are less likely to terminate a pregnancy and more likely to experience a birth outside marriage when they live in states with higher levels of economic inequality (Kearney and Levine 2014). The authors attribute this mechanism to the economic marginalization of women at the bottom of the income distribution, who live in states with high economic inequality, leading to desperation regarding socio-economic prospects and a declined motivation to avoid single motherhood among women with lower parental SES. However, this may be less true in contexts with lower levels of economic inequality, because the educational and economic prospects of those with lower and higher parental SES are more similar in more economically equal societies (Jerrim and Macmillan 2015). It is therefore possible that parental SES differences in the risk of conception for single women is less strong in more economically equal societies. Differences in economic inequality at the societal level can also influence parental SES differences in intergenerational financial or material transfers from parents to young adults (Albertini and Kohli 2013). In societies that are more economically equal, parental SES differences in entering a union during pregnancy may therefore be less strong.

## Method

### Data

Data from the first wave of the Generations and Gender Survey (GGS) version 4.2 were used to carry out the research for 15 countries (see Table 1) (Fokkema et al. 2016). GGS data on Australia, Italy, Japan, and the Netherlands were not used, because these countries provided insufficient information on fertility history, partnership history, or parental SES. We expanded our dataset by adding information from Canada, the USA, and the UK from other sources. For the USA and the UK, data from the Harmonized Histories (HH) taken from the National Survey of Family Growth and the British Household Panel Study were used (Perelli-Harris, Kreyenfeld, et al. 2010). For Canada, the General Social Survey—GSS—cycle 20 was used (Béchard and Marchand 2008).

**Table 1:** Descriptive information on the datasets used in this study

	Original dataset				Sample used for analyses				
	Source^a^	Collected	Age	Sample women	Total	Single upon conception (% of total)		Union transition (% of single upon conception)	
Austria	GGS	2008–09	18–46	3001	2941	370	(13%)	191	(52%)
Belgium	GGS	2008–10	18–82	3728	1854	130	(7%)	57	(44%)
Bulgaria	GGS	2004	17–85	7007	3998	560	(14%)	436	(78%)
Canada	GSS	2006	15–79	13262	5638	716	(13%)	222	(31%)
Czech Republic	GGS	2004–06	18–79	5209	2375	521	(22%)	340	(65%)
Estonia	GGS	2004–05	21–81	5034	2029	335	(17%)	188	(56%)
France	GGS	2005	18–79	5708	2439	145	(6%)	50	(34%)
Georgia	GGS	2006	18–80	5595	2688	131	(5%)	82	(63%)
Germany	GGS	2005	17–85	5407	2373	399	(17%)	144	(36%)
Hungary	GGS	2004–05	21–79	7517	2891	483	(17%)	364	(75%)
Lithuania	GGS	2006	17–80	5037	2198	443	(20%)	303	(68%)
Norway	GGS	2007–08	19–81	7541	3573	357	(10%)	160	(45%)
Poland	GGS	2010–11	18–84	11,578	5029	1263	(25%)	999	(79%)
Romania	GGS	2005	18–80	6009	2237	193	(9%)	148	(77%)
Russia	GGS	2004	17–81	7038	2557	417	(16%)	272	(65%)
Sweden	GGS	2012–13	18–80	4991	2630	151	(6%)	92	(61%)
UK	HH	2005–06	16–81	6101	2528	489	(19%)	141	(29%)
USA	HH	2006–08	15–45	7356	7148	1481	(21%)	418	(28%)

^a^*GGS* Generations and Gender Survey, *GSS* General Social Survey, *HH* Harmonized Histories

Combined, the datasets contain information of 117,119 women. We deleted women who were born before 1960 (*N*_deleted_ = 54,781) and who experienced their first conception prior to the age of 15 (*N* = 337). In addition, information was deleted for women with missing data on age at interview (*N* = 737), timing of first birth (*N* = 69), union status at pregnancy or birth of the first child (N = 931), or parental educational attainment (*N* = 3138). This resulted in an analytical sample of *N* = 57,126. Information on the original data set and the selected sample is provided in Table 1.

### Dependent Variables

The GGS and HH data contain retrospective information on the month and year of the start of all cohabitations and marriages. This was combined with information on month and year of the birth of the first biological child to establish union status (1) when a single woman experienced her first conception leading to a live birth and (2) during pregnancy. The Canadian GSS provides the year of data collection and the age of the respondent at which a certain event in the fertility and partnership histories occurred. The age is specified to one decimal place, allowing an accurate ordering of life events. We consider the start of the pregnancy to be eight months before the birth of the first child. This is a conservative strategy, the aim of which is to prevent incorrect assignment of pregnancies that occurred within a union as pregnancies that preceded one (Baizán et al. 2003). Fertility histories only include information on live births, and the implications of this are discussed in the discussion section.

### Parental SES

The GGS provides two indicators to capture parental SES: the educational and occupational level of the parents at age 15 of the respondent. In this study, parental educational attainment was assumed to be a better variable to capture parental SES. In some of the countries cited in the study, the generation of the parents was characterized by a high female participation in education, but much lower levels of female participation in the labour force. The use of information on parental education thus captures the data for both parents. Moreover, in a number of countries, information on parental occupation is not part of the survey.

Parental educational attainment combines information on the educational attainment of the respondent’s father and mother, by taking the mean value. Information on one of the parents was used where it was not available for both. Information on mother’s education was missing for 1.6% of the sample and on father’s education for 9.8% of the sample. We used the International Standard Level of Education (ISLED) coding system (Schröder 2014). Similar to the more commonly used International Standard Classification of Education (ISCED) coding system, ISLED is based on obtained diplomas and degrees. Differently from the categorical ISCED classification, ISLED is expressed as a continuous variable in the range 0–100. Because we converted the country-specific educational systems directly into ISLED, this resulted in a richer variable than would have been obtained with ISCED (Brons and Mooyaart 2018). Schröder (2014) provides country-specific information on the translation to ISLED for all countries in our dataset, except for Georgia, the USA, and Canada. For these countries, a general conversion scheme was used based on the correspondence between ISCED and ISLED in all countries of the European Social Survey (Schröder 2014). Parental SES was standardized into a country-specific z-score.

### Control Variables

The birth year of the respondent was included to control for cohort effects. To provide a more meaningful interpretation in the regression models, birth year was centred around the year 1970, which is close to the average birth year for the whole sample. For the models that estimate the likelihood of experiencing conception, age is entered as a categorical variable differentiating between 4-year periods between ages 15 and 30, as well as one extra category referring to age 31 + . For the models estimating the likelihood of continuing to live as a single woman during pregnancy, duration is entered as a categorical variable relating to (1) 6–8 months before childbirth, (2) 3–5 months before childbirth, (3) 1–2 months before childbirth, and (4) the month of birth of the first child.

### Macro-Indicators

Information on modern contraceptive use was obtained from the UN dataset ‘World Contraceptive Use 2017’ (Alkema et al. 2013). This dataset provides information on the prevalence of contraception, defined as the number of partnered women of reproductive age who are currently using modern contraception divided by the total number of partnered women of reproductive age. Information on abortion rates was acquired from the UN report ‘World Abortion Policies’ (United Nations 2007) and refers to the number of abortions per 1,000 women aged 15–44 years. Data on adolescent abortion rates were obtained from a study by Singh and Darroch (2000) and refer to abortion rates for women aged 15–19. Information on the adolescent abortion rate was not available for Austria, Lithuania, or Poland. Norms pertaining to marriage and single motherhood were obtained from the European Value Study and the World Value Survey (EVS 2011; WVS 2015). In these surveys, respondents were asked whether they agreed or disagreed with the statement *‘Marriage is an outdated institution’* and whether they approved or disapproved with the statement *‘If a woman wants to have a child as a single parent but she doesn't want to have a stable relationship with a man, do you approve or disapprove?’*. For each country, the proportion of the sample who agreed with each statement was calculated. A higher score thus means that the general population is more supportive of alternative family forms. For the item measuring approval of single motherhood, a third response option ‘*it depends*’ was available. These responses were not taken into account when calculating the overall proportion of respondents who approved of the statement. Economic inequality is captured with the GINI coefficient of economic inequality, obtained from The World Bank (http://iresearch.worldbank.org/PovcalNet/home.aspx).

For the macro-indicators, information between 1990 and 2010 was considered. The 1990 cut-off point was chosen because macro-level information is only available for a few countries prior to this time point. The cut-off point of 2010 was chosen because—with the exception of Sweden—micro-level information is not available after this year (see Table 1). Only for contraceptive use did we deem it important to extend the time-period to 1980–2010, in order to increase the number of data points. Because the number of data points fluctuates depending on the indicator and country considered, a line was fitted through the datapoints and the predicted value of the mean-point in the period (the year 2000) was used in the models. Abortion rates and adolescent abortion rates are only available for one year and refer to the information available closest to 2004 and 2000 respectively. All variables were standardized (into z-scores) before including them in the models. Online Supporting Materials provide an overview of the predicted values per country and indicator (Table A1), the Pearson correlations between macro-indicators (Table A2), and figures visualizing the datapoints and fitted line for each macro-indicator (Figures A1-A4).

### Analytical Strategy

To examine the influence of parental SES on the likelihood of experiencing conception while living as a single woman (H1), discrete-time event history logistic models of the monthly risk of experiencing a first conception were estimated. Women were followed from age 15 until the moment of conception or the moment of the interview. Women were removed from the risk set whenever they entered a union before experiencing conception; from this point onwards, they were no longer ‘at risk’ of experiencing a conception while living as a single person. The association between parental SES and the likelihood of entering a union during pregnancy (H2) was examined with logistic regressions using a similar strategy. Single pregnant women were followed from 8 months before birth until the moment they experienced a transition to union or until the moment of birth of their first child. All analyses were run separately for each country.

A consequence of using log-odds from logistic models is that, with increasing model fit, the scale of the dependent variable and hence the effect sizes of the independent variables tend to go up (Mood 2010). For the current study, this implies that differences in model fit between countries influence log-odds ratios of parental SES which makes the comparison of these effects across countries less reliable. Expressing the results in marginal effects instead of log-odds solves this problem (Mood 2010). In Stata, marginal effects can be obtained after a logistic regression with the post-estimation command *margin*. We have used this strategy to obtain the semielasticity, which provides information on the % change in Y given a 1-unit increase in X.

To assess whether the effect of parental SES on the outcome variables depend on the macro-indicators, the country-level effects of parental SES—expressed as semielasticities—were regressed on the country-level predictors. For this step, random effects meta-regressions were performed using the Knapp–Hartung modification. The models use weights which are based on the inverse of the standard error. In other words, more weight is given to estimates with a smaller confidence interval. Simulation studies have shown that this strategy can be used to provide reliable confidence intervals for linear and nonlinear models (Berkey et al. 1995; Higgins and Thompson 2004; Knapp and Hartung 2003). The method provides robust estimates as long as the number of datapoints at the macro-level is larger than five (Higgins and Thompson 2004). However, false positive rates can increase when more than one covariate is included at the macro-level (Higgins and Thompson 2004). Therefore, separate meta-analyses were run for each macro-indicator.

## Results

### Parental SES and Experiencing a Conception While Being Single

**Fig. 2:**
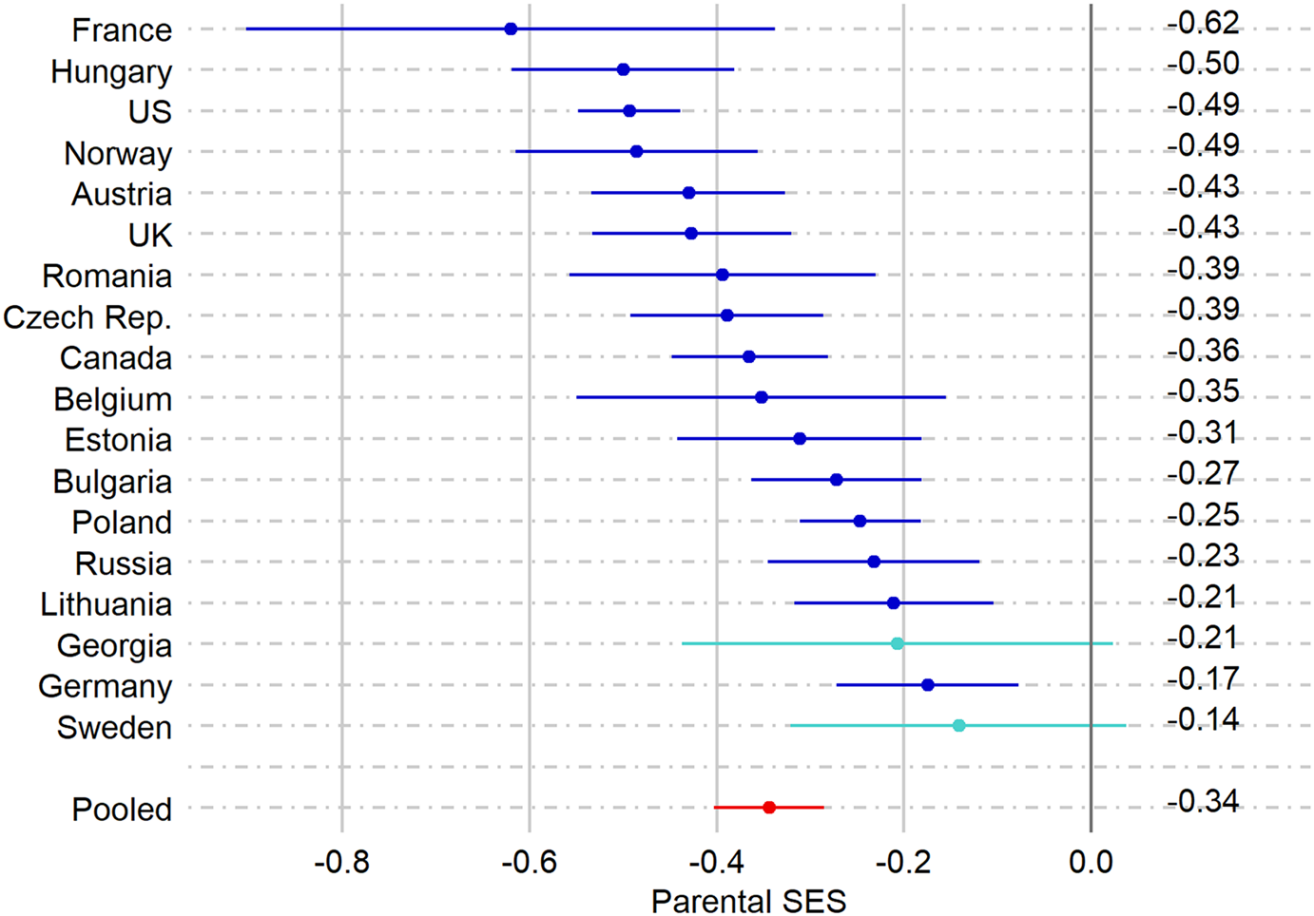
Country specific estimates (semielasticities) of associations of parental SES (expressed in z-scores) with the % change in probability of experiencing a conception among single women. The models are controlled for birth year and age

The results of the models of the association between parental SES and the probability of experiencing a first conception leading to a live birth while living as a single woman are shown in Fig. 2. The effects show the % change in the probability of experiencing a first conception given a one standard deviation increase in parental SES. Across all countries, a negative gradient of parental SES was found, with an effect size of: *b* = −0.34; 95-CI [− 0.40; − 0.29].^1^ In line with Hypothesis 1, single women with lower parental SES are more likely to experience conceptions than single women with higher parental SES. The largest effect was found for French women, their probability of experiencing a conception while single increases with 62% with a parental SES one standard deviation lower than the mean. The gradient did not reach statistical significance in Sweden and Georgia.

Online Supporting Materials provide information on effect sizes of the control variables in Table A5. Regarding the control variables, the analyses showed that experiencing a conception while living as a single woman is less common among more recent cohorts. Only in the USA has the likelihood of experiencing conception while single seen a statistically significant increase. Here, a clear linear effect of age was also found. Compared with single women aged 23–26, conception was more common among younger single women, and less common among older single women. In most other countries a curvilinear effect was found, in which compared to single women aged 23–26, younger single women (aged 15–18) and older single women (aged 31 +) were less likely to experience a conception.

Table A7 of Online Supporting Materials shows the results of additional models including an interaction term between age and parental SES. The analyses reveal that the effect of parental SES on the likelihood to experience a conception while being single is more negative at age 15–18 than at age 23–26 in Georgia, Hungary, Romania, Bulgaria, and Lithuania. In Hungary and Lithuania, the effect of parental SES on the dependent variable is also more negative at age 19–22 than at age 23–26. The effect of parental SES on the likelihood to experience a conception while being single is more positive at age 31+ than at age 23–26 in France, the UK, Poland, and Sweden.

### Parental SES and Starting a Union during Pregnancy

**Fig. 3:**
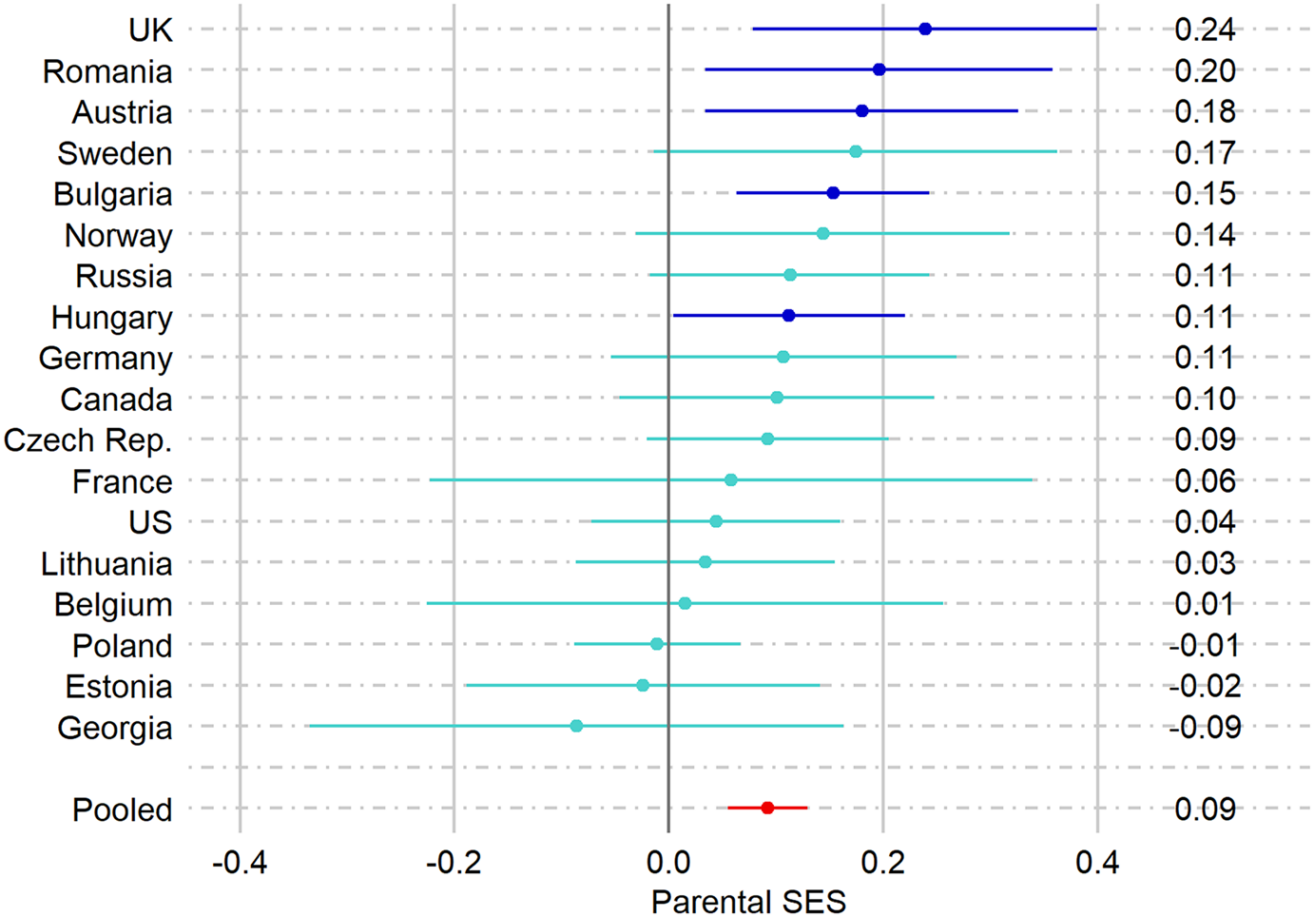
Country specific estimates (semielasticities) of associations of parental SES (expressed in z-scores) with the % change in probability of starting a union during pregancy. The models are controlled for birth year and age

In the next step, the association between parental SES and the probability of starting a union during pregnancy was examined (Fig. 3). Across all countries, the overall effect reached statistical significance: *b* = 0.09; 95-CI [0.06; 0.13].^2^ In line with Hypothesis 2, single women with lower parental SES are less likely to enter a union during pregnancy than single women with higher parental SES. The largest effect was found in the UK. Here, the probability of starting a union during pregnancy was 24% higher for women with parental SES one standard deviation higher than the mean. A significant association of parental SES on the probability of starting a union during pregnancy was also found in Romania, Austria, Bulgaria, and Hungary. In all other countries, no significant association was found with parental SES. In the case of Sweden, with its relatively large effect size, the non-significant effect may be related to the small sample size (see Table 1).

Online Supporting Materials provide information on effect sizes of the control variables in Table A6. The analyses reveal that in four Central European countries—Romania, the Czech Republic, Poland, and Estonia—the likelihood of starting a union during pregnancy is smaller for more recent cohorts. Generally, women are more likely to make the transition to a union after the first trimester of the pregnancy.

### Explaining Cross-National Differences in the Association of Parental SES

Thus far, the analyses have revealed that single women with lower parental SES are more likely to experience conceptions. Figure 2 illustrates that a substantial variation in the magnitude of this association across countries is found. Further inspection of the effects shows that about 81% of the variance in the association of parental SES can be attributed to between-country variation. In the next step, we examined whether macro-indicators could explain between-country variation in the association of parental SES.

**Fig. 4:**
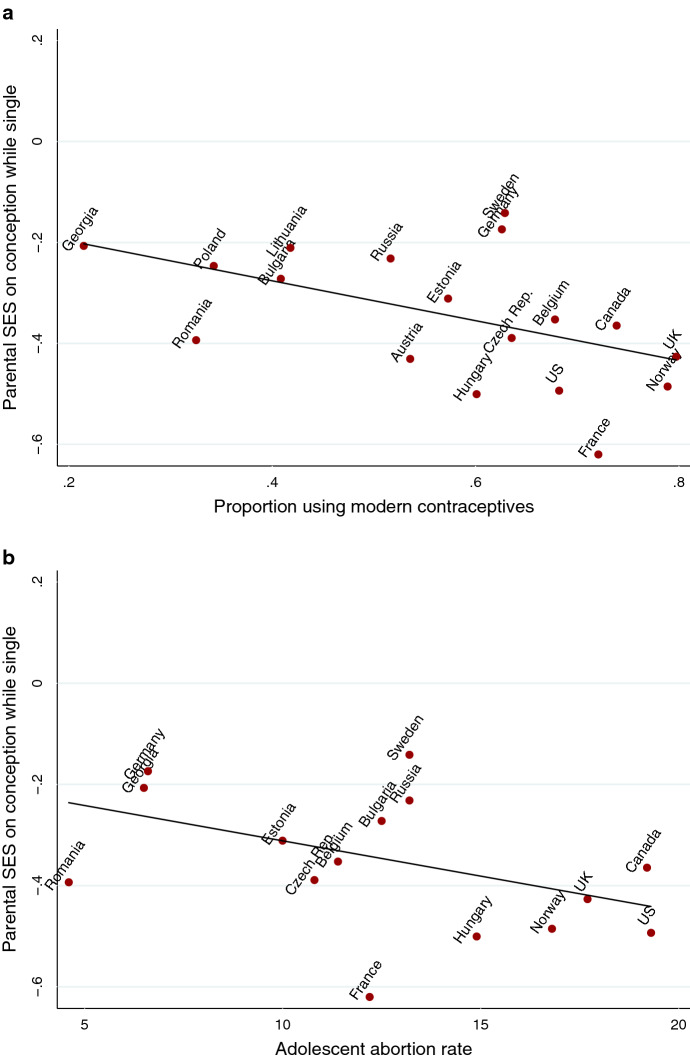
Results of the meta-regression showing the association between parental SES and the probability of experiencing a first conception while single, by modern contraceptive use and adolescent abortion rate

Results from the meta-regressions are presented in the second column of Table 2. The strength of the association between parental SES and experiencing conception while single depend significantly on the country’s level of modern contraceptive use and adolescent abortion rates. These associations are presented in Fig. 4. Although a negative association of parental SES with conceptions by single women was found in all countries, Fig. 4 shows that this association is twice as strong in countries with higher levels of contraceptive use and adolescent abortion rates.

**Table 2:** Results of the meta-regression showing the associations of the macro-indicators with the effect of parental SES on the probability of experiencing a conception while single (column 2) and of starting a union during pregnancy (column 3)

	Parental SES on conception while single		Parental SES on starting a union during pregnancy	
Modern contraceptive use	− .07 (.03)*		.03 (.02)	
Abortion rate	.01 (.03)		.01 (.02)	
Adolescent abortion rate	− .06 (.03)*		.00 (.02)	
Less conservative marriage norms	− .01 (.03)		.05 (.02)*	
Less conservative single mother norms	.03 (.03)		− .01 (.02)	
Economic inequality	.01 (.03)		− .02 (.02)	

The effect of parental SES is expressed in semielasticity, providing information on the % change in the dependent variables, given a 1-unit increase in parental SES. Parental SES and the macro-indicators are expressed in z-scores

***p < .001; ** *p* < .01; *p < .05

Although it was expected that abortion rates among women aged 15–44 and economic inequality could influence the association between parental SES and experiencing a conception while single, Table 2 shows that these interactions are not statistically significant. Because the meta-regressions are based on 18 data points, it is possible that this is due to the low statistical power of the model. Figure A6 of Online Supporting Materials provides an overview of all interaction effects of the macro-indicators. Inspection of the figure of economic inequality and abortion rates of women aged 15–44 reveals that there are no reasons to suspect that low statistical power has caused the non-significant interaction effects. Russia appears to be an outlier when it comes to the influence of abortion rate on the association of parental SES with conceptions while being single. However, a non-significant interaction effect is still found after leaving this data point out of the meta-regression.

**Fig. 5:**
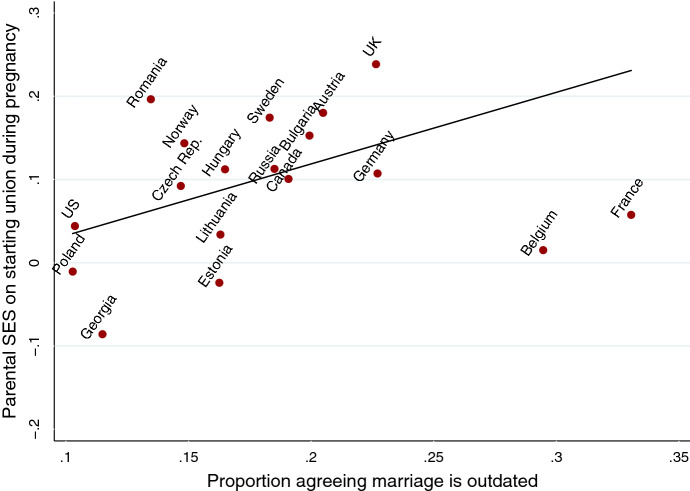
Results of the meta-regression showing the association between Parental SES and the probability of starting a union during pregnancy, by marriage norms

Overall, the between-country variation in the association between parental SES and the likelihood of making a transition to a union during pregnancy was relatively small (22%). We did find a significant positive effect of a country’s norms regarding marriage on the influence of parental SES (see column 3 of Table 2). The association is presented in Fig. 5 and shows that the positive association between parental SES and the likelihood of starting a union was only found in countries with less conservative norms regarding marriage. The analyses showed no parental SES gradient in Belgium and France, even though these countries scored highest in terms of the proportion of the population who agreed with the statement that marriage is outdated. These countries could be coincidental outliers; however, it is also possible that this is the first sign of a curvilinear relationship.

Against expectation, no statistically significant effects were found of norms regarding single motherhood and economic inequality on the association of parental SES with the likelihood of making a transition to a union during pregnancy. After a visual inspection of these interaction effects with the help of Figure A7 of Online Supporting Materials, we deem it unlikely that the insignificant interaction effect of norms regarding single motherhood is the result of low statistical power of the model. We cannot completely rule out that a model with more statistical power would find a significant effect of economic inequality on the association of parental SES with the likelihood of a union transition.

One may argue that the interaction effects of contraceptive use and adolescent abortion rate on the association of parental SES with the likelihood of experiencing a first conception while living as a single woman, merely reflect a spurious effect through general development in a country. We therefore also entered the Human Development Index of each country in the models. HDI consists of normalized indices in three dimensions: long and healthy life, knowledge, and a decent standard of living, and captures general levels of development in a country. Although HDI is moderately correlated with teen abortion rate with r = 0.58 and strongly correlated with modern contraceptive use with r = 0.86, no significant association of HDI was found with the gradient of parental SES. This supports our belief that the effect found for family planning is not a spurious effect of general development. Online Supporting Materials provide information on the predicted values of HDI per country (Figure A5), as well as the Pearson correlation with other macro-indicators (Table A3) and the outcomes of the meta-regressions (Table A4).

## Discussion

Western societies have been confronted with an increasing disconnection between marriage and childbearing. The increase in the number of single-parent families has given rise to concerns, because poverty rates are much higher among these families than among two-parent families. This is especially true for families of women who have been living without a partner since the start of motherhood. The current study focuses on understanding the association between parental SES and the likelihood of women experiencing a first birth while single and identifying societal factors influencing this association. This was examined for 18 European and North American countries.

Our first question was at what stage in family formation do differences in parental SES come about? We hypothesized that conceptions leading to live births are more common among single women with lower parental SES (H1) and that women with lower parental SES are less likely to enter a union during pregnancy (H2). In addition, we examined whether there are cross-national differences in the association of parental SES and to what extent these differences can be explained by a country’s access to family planning, support of alternative forms of family formation, and level of economic inequality.

Overall, we found a significant negative gradient of parental SES with the probability of experiencing a first conception while living single, which confirms our first hypothesis. The literature points to two plausible mechanisms to explain this finding. It is possible that women with higher parental SES take more care to prevent pregnancy, for example because they are concerned about their educational and occupational prospects. Women with lower parental SES might not necessarily plan to become a single mother, but might be more ambivalent about the subject and might welcome motherhood. An alternative—and perhaps more worrying—possibility is that, regardless of parental SES, women could be equally motivated to prevent single motherhood, but women with lower parental SES may lack the ability to do so.

The analyses also revealed substantial cross-national variation in the association of parental SES and the likelihood that single women experience conceptions. We believed that societal differences in access to family planning could influence parental SES difference in the likelihood that single women would experience conception. However, it was less clear which direction this effect would take. One could argue that in societies where access to family planning is limited, women with higher parental SES have the means to access it due to the availability of material and financial resources, whereas this effect disappears when modern contraceptives are more readily available to the wider public. However, the results showed that the association between parental SES and the likelihood of experiencing a conception among single women is stronger in countries with higher modern contraceptive use and higher adolescent abortion rates. Even though the Human Development Index was moderately correlated with adolescent abortion rate and strongly correlated with modern contraceptive use, no significant association of HDI was found with the gradient of parental SES with the likelihood that single women experience conceptions. This strengthens our belief that the association of family planning is not based on a spurious effect. The results therefore provide a first indication that in countries with wider access to family planning, women with higher parental SES make more use of these methods. This could be due to the more severe consequences of becoming a single parent for this group. As one of the reviewers pointed out: ‘Kingsley Davis used to say, family planning is like an airline ticket; it’s only useful if you want to go somewhere’. An alternative explanation, which is not commonly mentioned in the literature on single motherhood, is that women with lower parental SES are perhaps more religious and are therefore reluctant to use available family planning methods. This mechanism could particularly play a role in more religious societies (Schwadel 2015).

A more thorough examination of the relationship between different types of family planning methods and socio-economic differences in the likelihood of becoming a single mother is perhaps possible in future research, by making use of data which holds information on pregnancies that have not been brought to term. However, this approach would come at the expense of datasets that can be used and countries that can be examined, especially in Europe. Further insight can be gained from an examination of the effect of family planning methods on parental SES differences in single motherhood within countries over time. In this case, research could benefit from a focus on a context where detailed information on family planning methods and policies is available for a long period of time. This should preferably be linked with a large, perhaps register-based, individual-level sample to maintain analytical power. For an example, see the research of Schneider and Gemmill (2016) who used this approach to explain racial/ethnic differences in non-marital fertility rates over time in the USA.

Abortion rates of women aged 15–44 years were not found to have a significant interaction effect. Partnered women may also abort because they do not want an extra child. It is therefore probable that adolescent abortion rates reflect abortion rates among childless single women better, and are therefore a better predictor in our models. We also believed that the level of economic inequality in a country could influence the association between parental SES and the likelihood of single women becoming pregnant. However, no interaction effect was found with the level of economic inequality in a country. This contrasts with findings of Kearney and Levine (2014), who showed that in the USA, young women with lower parental SES are less likely to terminate a pregnancy and more likely to experience a non-marital birth when they live in more unequal communities. The authors attributed this to more economic marginalization and desperation among women at the bottom of the income distribution, who live in areas where economic inequality is high. Perhaps economic inequality only influences early births, whereas our study examined first births to single women of all ages. In line with previous research (Smith et al. 2018), our analyses show that the US context is unique in comparison with other Western societies. It is the only country in the sample where conceptions were more common among single women aged 15–18 than among those aged 23–26, and the only country where the likelihood that single women would experience a conception significantly increased for more recent cohorts. This unique context may explain why the findings for the USA are not generalizable to other Western societies.

Overall, a positive gradient of parental SES was found on the likelihood of single women entering a union during pregnancy, which confirms our second hypothesis. Some degree of caution is required when interpreting this association. It is possible, for example, that couples reduce contraceptive use in anticipation of marriage or cohabitation. In this case, the parental SES gradient may reflect the simple fact that women with higher parental SES are more likely to reduce contraceptive use in anticipation of a union compared to women with lower parental SES. However, in most countries, women were more likely to make a transition to a union after the first trimester of the pregnancy, e.g., during the period when people generally find out about a pregnancy. The more plausible interpretation is therefore that women with higher parental SES are more likely to enter a union in reaction to a pregnancy than women with lower parental SES.

The positive association between parental SES and the likelihood of women entering a union during pregnancy was only found in countries with less conservative norms regarding marriage. We must leave it to future research to examine the reasons for this. It is possible that in societies more open to alternative forms of family, single motherhood is less stigmatized. This may influence women with lower parental SES more, because they perceive the prospect of becoming a single mother as less detrimental. The results furthermore show that the effect size was substantially supressed by two countries: Belgium and France. While these countries have the highest proportion of their populations who agree with the statement that marriage is outdated, the parental SES gradient with the probability of enter a union during pregnancy in these countries was not present. Future research, perhaps using more recent data, should clarify whether these are coincidental outliers, or if this is instead the first sign of a curvilinear relationship, where less conservative norms in society only temporarily induce a positive parental SES gradient on the likelihood of entering a union during pregnancy.

Perhaps surprisingly, the association between parental SES and the likelihood of entering a union during pregnancy was not influenced by the extent to which the population is supportive of single motherhood. The reason for this remains unclear. However, the low correlation at the country level between the items reflecting marriage norms and norms regarding single motherhood suggests that they capture different forms of support for alternative behaviours in terms of family formation. Single motherhood is perhaps often viewed as a consequence of separation from a partner, and views regarding single motherhood may explain parental SES differences in the likelihood of divorce in a country better than of entering a union. We believed that economic inequality in a country could also influence the association between parental SES and the likelihood that women would enter a union during pregnancy. Resources invested by parents with lower and higher SES in their children are more similar in more economically equal societies, which could reduce the negative gradient of parental SES on the probability of entering a union during pregnancy. However, the results did not support this notion.

Previous research has shown that women with low parental SES tend to enter their union earlier than women with high parental SES (Brons et al. 2017; Sassler et al. 2018). This leaves women with low parental SES less exposed to single motherhood. A disadvantage of the methodological approach used in this study is that it does not control for the influence of parental SES on overall union transition rates. It is therefore possible that the negative effects of parental SES on the dependent variables estimated in this study are too conservative and a stronger effect of parental SES will be found if the transition to a union and that to a birth are modelled simultaneously. In addition, it is important to stress that the current study examined conceptions which led to a live birth. Because single women with lower parental SES are perhaps more likely to have a spontaneous or induced abortion than single women with higher parental SES (E. Carlson et al. 1999; Norsker et al. 2012), a stronger effect of parental SES on conception rates could arise for single women when all pregnancies are taken into account. Lastly, we would like to point out that in this study the influence of only three societal factors was tested. We will have to leave it to future research to establish if other societal factors, such as level of religiosity and inequality in educational opportunities, can explain cross-national variation in the association of parental SES with single motherhood.

To conclude, this study contributes to the literature by showing that the parental SES gradient present at the start of single motherhood mostly lies in a higher probability of women with lower parental SES to experience a conception outside a union. In some societies, the parental SES gradient is aggravated during pregnancy, because women with lower parental SES are less likely to start living with a partner at this point. The negative gradient of parental SES on single motherhood was stronger in countries with better access to family planning, and in societies which are more supportive of alternative forms of family formation. The results suggest that general developments in Western societies may not be beneficial to all, and may increase parental SES differentiation in family demography. Many Western societies have policies in place to combat the negative socio-economic consequences of single parenthood. The results of this study suggest that policies that aim to reduce the unequal use of family planning methods may be beneficial too, because they may reduce the inequality already present at the start of single motherhood.

## Supplementary Information

Below is the link to the electronic supplementary material.

Supplementary file1 (PDF 907 kb)

## Data Availability

This article is based on data that are freely available to researchers on the conditions that the researchers in question are attached to a recognized research institute and the data are not used for commercial purposes.

## Footnotes

1 Additional analyses reveal that the assumption of a linear effect of parental SES on the likelihood of single women experiencing a conception holds in the majority of countries. Only in Georgia, France, Hungary, Estonia, Poland, and the US, a significant effect of the quadratic term of parental SES was found.

2 Additional analyses reveal that the assumption of a linear effect of parental SES on the likelihood of starting a union during pregnancy holds in the majority of countries. Only in the US, a significant effect of the quadratic term of parental SES was found.

## Code availability

The code is stored at the Netherlands Interdisciplinary Demographic Institute. In case of doubt about the replicability of the results of this article, the Netherlands Interdisciplinary Demographic Institute can be contacted.
