# Supplementary material for: Socio-Economic Differences in the Prevalence of Single Motherhood in North America and Europe
Source: Eur J Popul. 2021 Jul 13;37(4-5):825–49. doi: 10.1007/s10680-021-09591-3 (PMC8575729; doi:10.1007/s10680-021-09591-3)
Supplement: Supplementary file 1 — Supplementary file1 (PDF 907 kb) [file 10680_2021_9591_MOESM1_ESM.pdf]

ONLINE SUPPORTING INFORMATION

SOCIO-ECONOMIC DIFFERENCES IN THE PREVALENCE OF SINGLE MOTHERHOOD IN NORTH-AMERICA AND EUROPE

Table A1. *Overview of the Macro-Level Indicators per Country.*

	Modern contraceptive use	Abortion rate	Adolescent abortion rate	Proportion agreeing marriage is outdated	Proportion approving of single motherhood	GINI economic inequality
Austria	.54	1.3	n.a.	.20	.58	30
Belgium	.68	7.5	11.4	.29	.56	32
Bulgaria	.41	21.3	12.5	.20	.59	30
Canada	.74	15.2	19.2	.19	.57	33
Czech Rep.	.64	12.2	10.8	.15	.60	26
Estonia	.57	33.3	10.0	.16	.59	35
France	.72	16.9	12.2	.33	.64	32
Georgia	.21	19.1	6.5	.11	.62	40
Germany	.63	7.8	6.6	.23	.53	30
Hungary	.60	23.4	14.9	.16	.53	28
Lithuania	.42	13.9	n.a.	.16	.80	33
Norway	.79	15.2	16.8	.15	.44	27
Poland	.34	0.0	n.a.	.10	.46	32
Romania	.33	27.8	4.6	.13	.62	29
Russia	.52	53.7	13.2	.19	.64	41
Sweden	.63	20.2	13.2	.18	.47	26
UK	.80	17.0	17.7	.23	.47	37
US	.68	20.8	19.3	.10	.52	40

NOTE: All values refer to the year 2000. For abortion rate and adolescent abortion rate it refers to information available closest to 2000 and 2004 respectively.

Table A2. *Pearson Correlations between Macro-Level Indicators.*

	Modern contraceptive use	Abortion rate	Adolescent abortion rate	Marriage norms	Single mother norms
Abortion rate	-.08				
Adolescent abortion rate	.71	-.03			
Less conservative marriage norms	.48	-.11	.01		
Less conservative single mother norms	-.43	.25	-.53	.11	
Economic inequality	-.16	.43	.15	-.13	.21

NOTE: Information on adolescent abortion rate is not available for Austria, Lithuania, and Poland. For this macro-indicator the Pearson correlations are estimated for 15 countries.

FIGURE A1. GRAPH, SHOWING, PER COUNTRY, THE DATAPOINTS AND FITTED LINE FOR THE PROPORTION OF PARTNERED WOMEN OF REPRODUCTIVE AGE USING MODERN CONTRACEPTIVES.

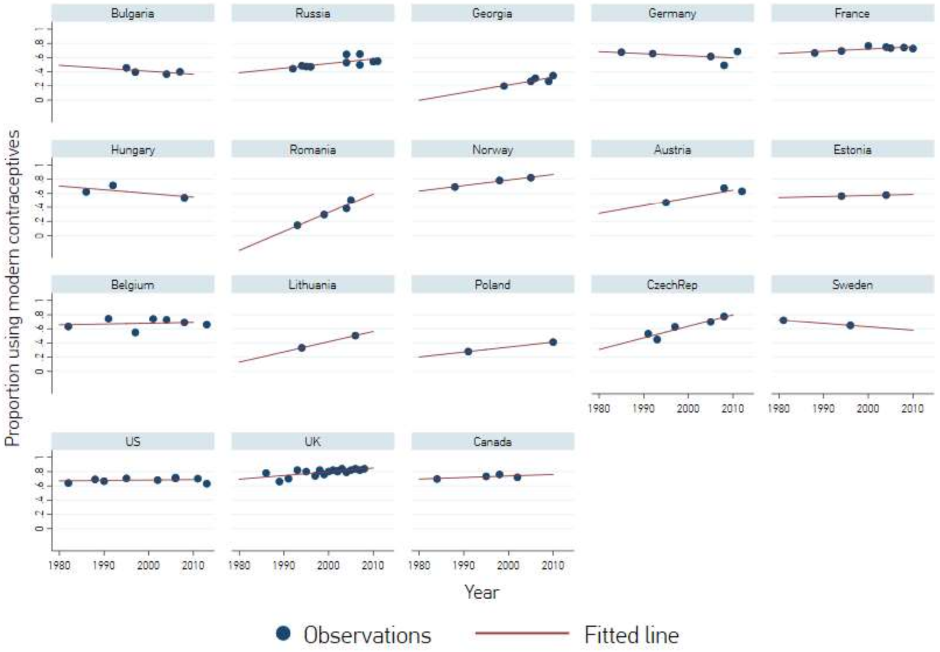

FIGURE A2. GRAPH, SHOWING, PER COUNTRY, THE DATAPOINTS AND FITTED LINE FOR THE PROPORTION AGREEING THAT MARRIAGE IS AN OUTDATED INSTITUTION.

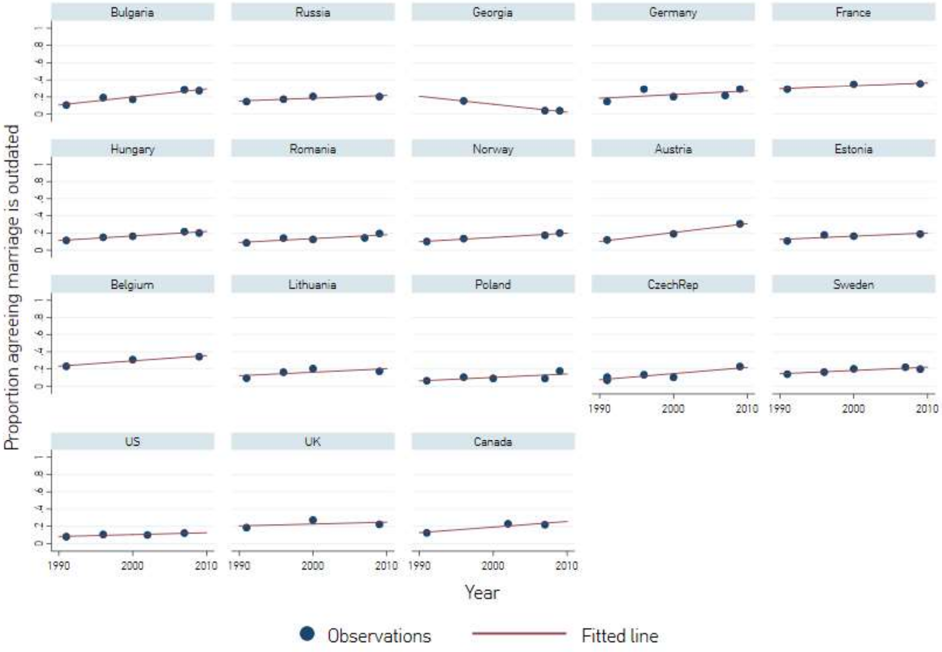

FIGURE A3. GRAPHS, SHOWING, PER COUNTRY, THE DATAPOINTS AND FITTED LINE FOR THE PROPORTION APPROVING OF SINGLE MOTHERHOOD.

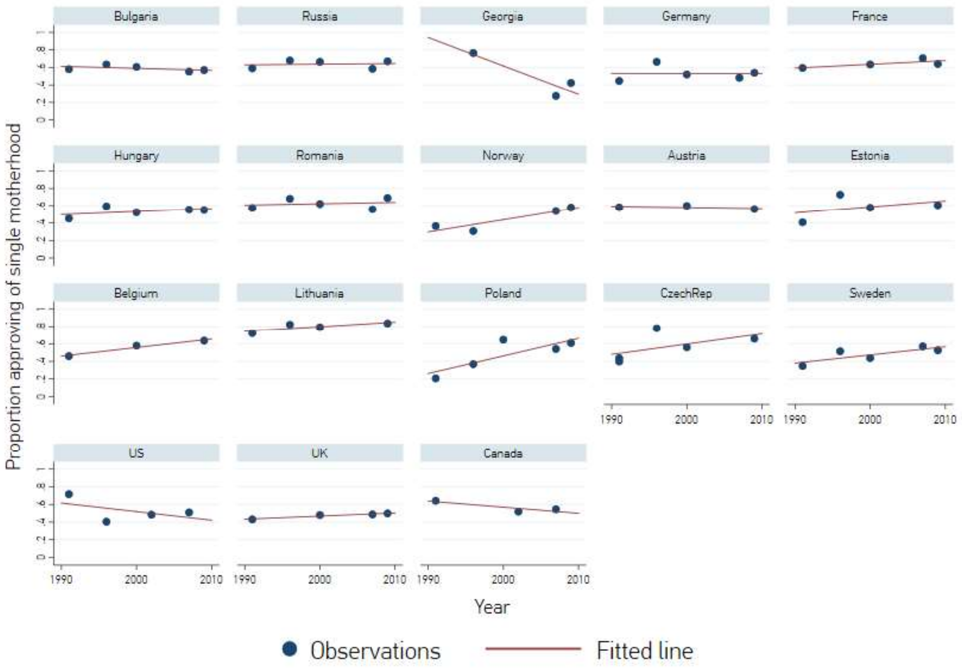

FIGURE A4. GRAPHS, SHOWING, PER COUNTRY, THE DATAPOINTS AND FITTED LINE FOR THE GINI COEFFICIENT OF ECONOMIC INEQUALITY.

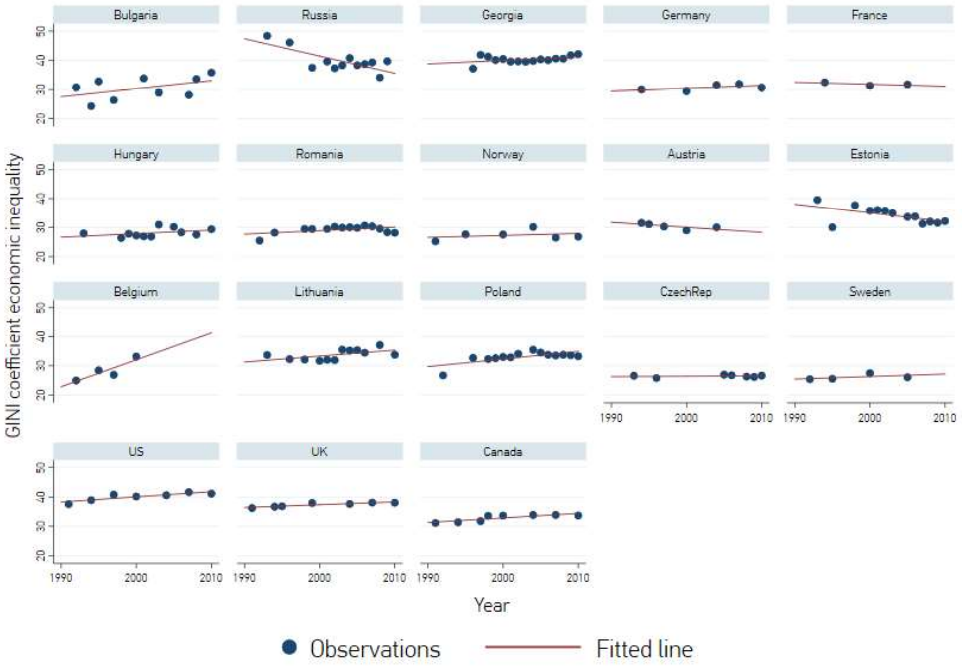

FIGURE A5. GRAPHS, SHOWING, PER COUNTRY, THE DATAPOINTS AND FITTED LINE FOR THE HUMAN DEVELOPMENT INDEX.

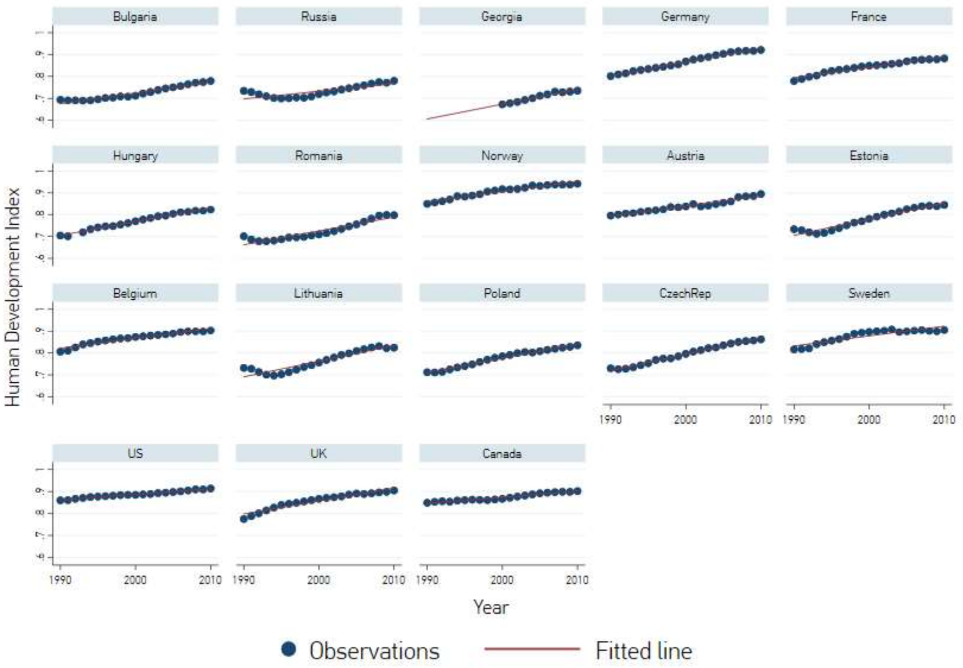

Table A3. *Pearson Correlations between the Human Development Index (HDI) and Other Macro-Level Indicators.*

	Modern contraceptive use	Abortion rate	Adolescent abortion rate	Less conservative marriage norms	Less conservative single mother norms	Economic inequality
HDI	.86	.40	.58	.35	-.54	-.25

NOTE: Information on adolescent abortion rate is not available for Austria, Lithuania, and Poland. For this macro-indicator the Pearson correlations are estimated for 15 countries.

Table A4. *Results of the Meta-Regression Showing the Associations of the Human Development Index (HDI) with the Association of Socio-Economic Background on the Probability of Experiencing a Conception while Single (Column 2) and of Starting a Union during Pregnancy (Column 3). Parental SES and HDI are Expressed in Z-Scores.*

	SE background on conception while single	SE background on continuing to live single during pregnancy
HDI	-.05 (.03)	.01 (.02)

*** $p<.001$; ** $p<.01$; * $p<.05$

Table A5. *Country Specific Estimates (Log-Odds) of Associations of the Individual-Level Indicators with the Likelihood of Experiencing a Conception among Single Women. Parental SES is Expressed in Z-Scores.*

	Parental SES	Birth year (ref. 1970)	Age (ref. 23-26) 15-18	19-22	27-30	31+	Constant
France	-0.62*	-0.03	-0.49***	-0.03	0.14	-2.18**	-7.19*
Hungary	-0.50*	-0.06*	-0.26	0.40**	-0.39	-1.67*	-6.05*
US	-0.50*	0.01*	0.42*	0.51*	-0.19	-0.93*	-6.54*
Norway	-0.49*	-0.06*	-0.62*	0.05	-0.17	-1.38*	-6.64*
Austria	-0.43*	-0.05*	-0.40***	0.33***	-0.22	-1.39*	-6.61*
UK	-0.42*	0.01	0.02	0.36***	-0.39	-1.14*	-6.36*
Romania	-0.39*	-0.03***	0.09	0.37	-0.63	-1.10***	-6.81*
Czech Rep.	-0.39*	-0.08*	-0.65*	0.42**	-0.81**	-2.70*	-5.68*
Canada	-0.37*	0.01	-0.18	0.14	-0.40***	-0.99*	-6.69*
Belgium	-0.35*	-0.03***	-1.23*	-0.40	0.17	-1.03**	-6.81*
Estonia	-0.31*	-0.06*	-0.51**	0.58*	0.12	-0.84***	-6.16*
Bulgaria	-0.27*	-0.03*	-0.40**	0.21	-0.78**	-1.31*	-6.02*
Poland	-0.25*	-0.02*	-0.66*	0.39*	-0.84*	-1.70*	-5.64*
Russia	-0.23*	< -0.01	-0.67*	0.31***	-0.37	-1.20**	-5.87*
Lithuania	-0.21*	-0.02*	-0.90*	0.18	-0.48	-1.25*	-5.70*
Georgia	-0.21	-0.01	-0.67**	0.03	-0.68	-1.77**	-7.11*
Germany	-0.17*	-0.02***	-0.94*	-0.01	0.07	-1.09*	-6.15*
Sweden	-0.14	-0.05*	-1.22*	0.01	0.19	-0.11	-7.15*

*** *p*<.001; ** *p*<.01; * *p*<.05

Table A6. *Country Specific Estimates (Log-Odds) of Associations of the Individual-Level Indicators with the Likelihood of Starting a Union during Pregnancy. Parental SES is Expressed in Z-Scores.*

	Parental SES	Birth year (ref. 1970)	Duration (ref. 6-8 months before birth) 3-5m before birth	1-2m before birth	Month of birth	Constant
UK	0.25**	-0.01	0.85*	0.91*	0.84**	-3.91*
Romania	0.23***	-0.04***	0.60**	0.18	0.65	-2.13*
Austria	0.19***	-0.01	1.07*	0.64**	1.12*	-3.12*
Sweden	0.19	0.02	0.71**	0.55	0.19	-2.68*
Bulgaria	0.18*	-0.01	0.66*	-0.34	-1.35**	-1.87*
Norway	0.15	-0.03	0.55**	-0.12	-0.07	-2.90*
Hungary	0.13***	-0.01	0.94*	0.08	-0.30	-2.18*
Russia	0.13	< -0.01	0.48*	-0.42	-0.22	-2.22*
Germany	0.11	-0.01	0.57**	0.38	-0.50	-3.27*
Canada	0.11	-0.02	0.04	-0.47***	0.43***	-3.15*
Czech Rep.	0.10	-0.03**	0.77*	-0.54***	-0.33	-2.30*
France	0.06	< -0.01	-0.37	-0.56	-0.15	-2.79*
US	0.05	-0.01	0.22	-0.21	-0.01	-3.30*
Lithuania	0.04	< -0.01	0.88*	0.15	-0.82	-2.34*
Belgium	0.02	0.01	1.07*	-0.47	0.29	-3.20*
Estonia	-0.03	-0.04**	0.42***	-0.24	-0.62	-2.44*
Poland	-0.01	-0.02*	1.74*	1.13*	0.81*	-2.71*
Georgia	-0.10	-0.01	0.33	-0.14	0.37	-2.27*

*** *p*<.001; ** *p*<.01; * *p*<.05

FIGURE A6. RESULTS OF THE META-REGRESSION SHOWING THE ASSOCIATIONS OF THE MACRO-INDICATORS WITH THE EFFECT OF PARENTAL SES ON THE PROBABILITY OF EXPERIENCING A CONCEPTION WHILE SINGLE.

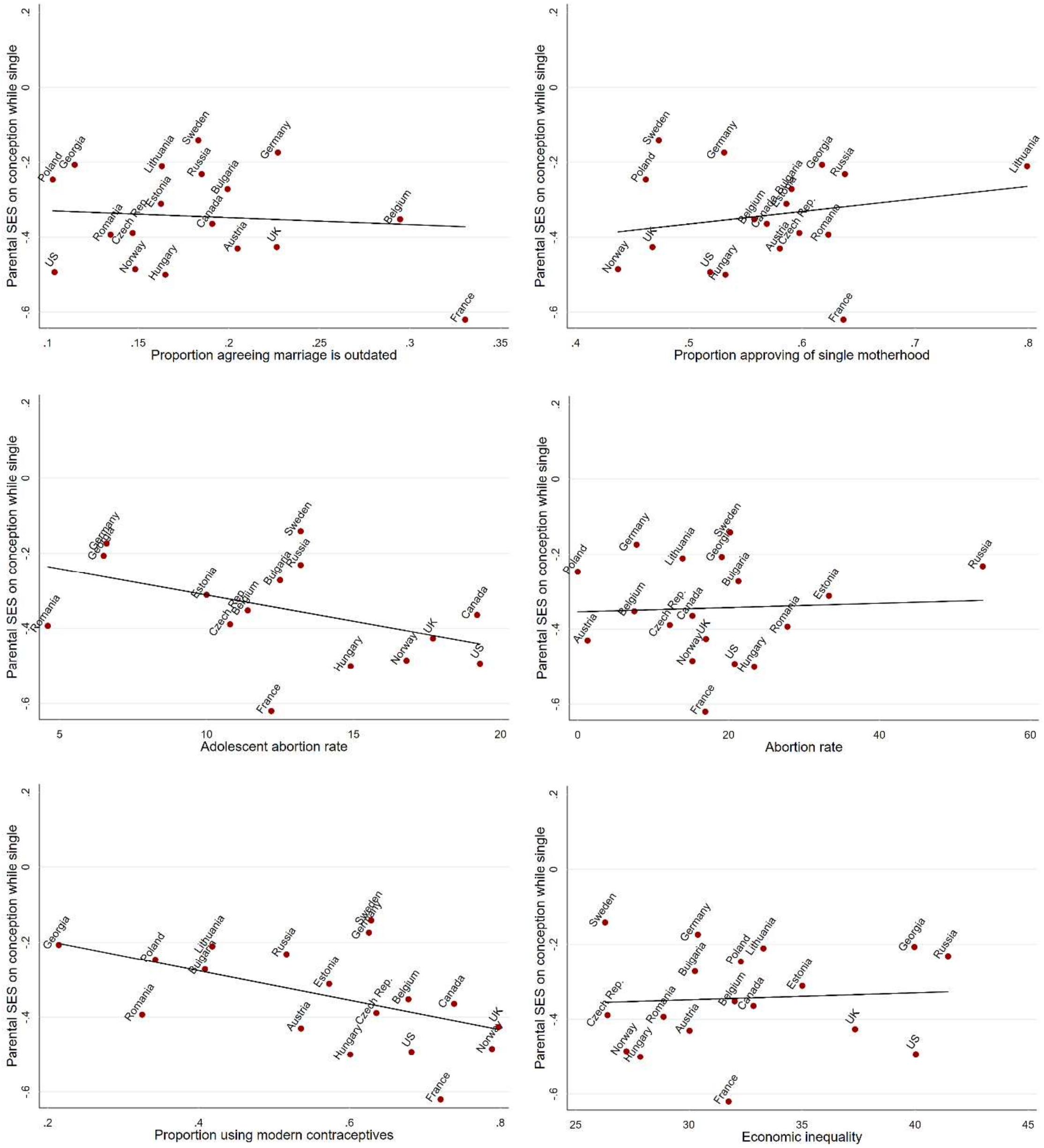

FIGURE A7. RESULTS OF THE META-REGRESSION SHOWING THE ASSOCIATIONS OF THE MACRO-INDICATORS WITH THE EFFECT OF PARENTAL SES ON THE PROBABILITY OF STARTING A UNION DURING PREGNANCY.

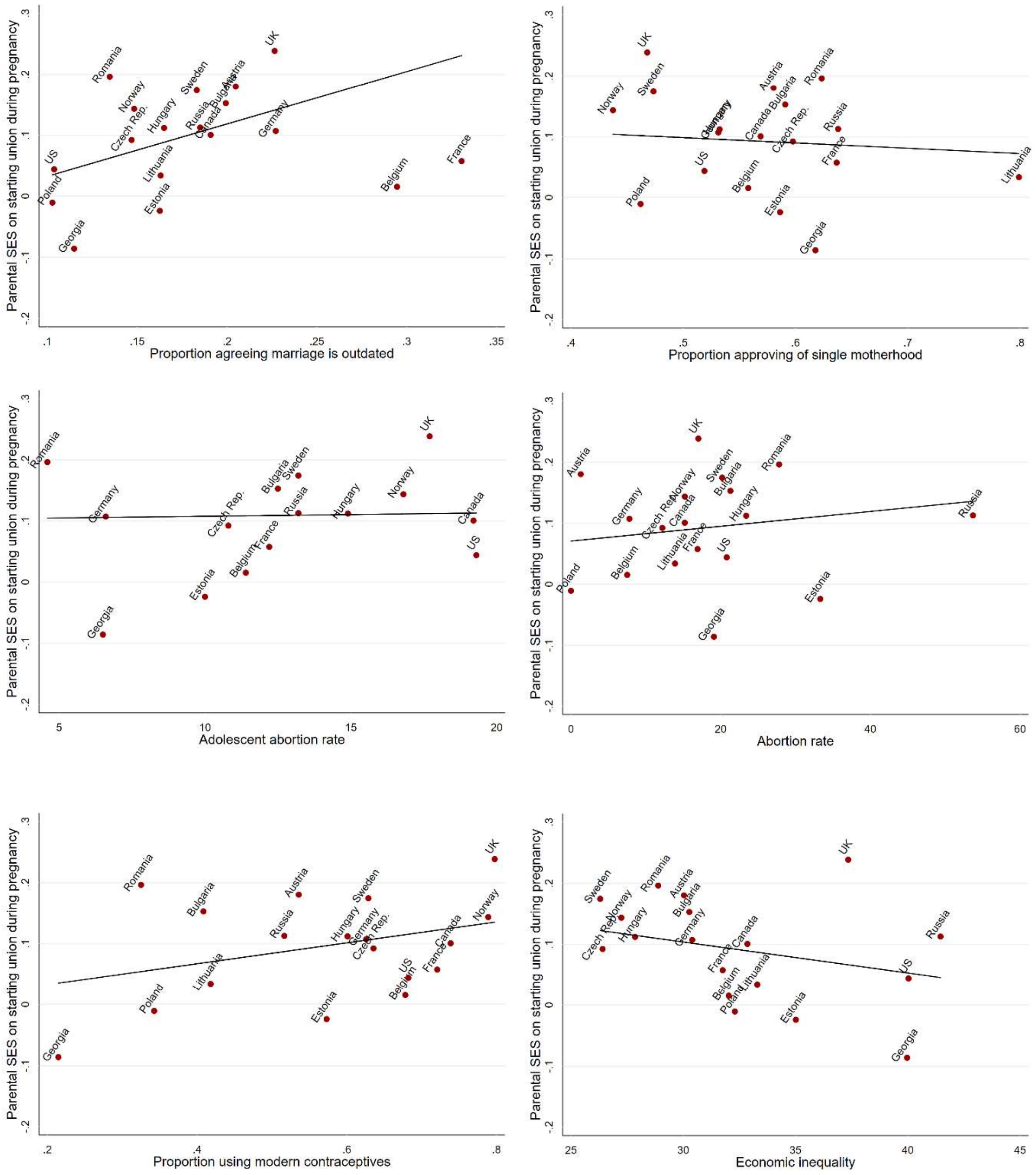

Table A7. *Country Specific Estimates (Log-Odds) of Associations of the Individual-Level Indicators with the Likelihood of Experiencing a Conception among Single Women. Parental SES is Expressed in Z-Scores.*

	Interaction Parental SES with Age				Parental SES	Age (ref. 23-26)				Birth year	Constant
	15-18	19-22	27-30	31+		15-18	19-22	27-30	31+		
Georgia	-0.49**	-0.01	-0.06	-1.13	0.07	1.59***	0.10	-0.33	2.57	-0.01	-7.54*
Hungary	-0.47*	-0.25**	0.05	0.10	-0.06	1.61*	1.50*	-0.54	-1.89***	-0.05*	-6.01*
Romania	-0.42***	-0.26	0.31	0.13	-0.06	1.47***	1.29	-1.86	-1.46	-0.03***	-6.82*
Bulgaria	-0.36*	-0.13	-0.16	0.06	0.01	1.04**	0.78***	-0.08	-1.47	-0.03*	-6.22*
Lithuania	-0.19***	-0.16***	-0.10	0.08	0.00	-0.09	0.89***	-0.02	-1.52***	-0.02*	-5.79*
France	-0.14	-0.11	0.33	0.82***	-0.38	-0.09	0.27	-0.94	-5.45**	-0.02	-5.93*
UK	0.08	0.05	0.08	0.66**	-0.39*	-0.31	0.14	-0.74	-4.22*	0.01	-4.64*
Poland	-0.07	-0.05	-0.10	0.46*	-0.13***	-0.36	0.61***	-0.44	-3.71*	-0.02*	-5.15*
Sweden	-0.21	-0.06	0.31	0.42***	-0.15	-0.34	0.26	-1.38	-2.17***	-0.05*	-6.46*
Canada	-0.05	-0.10	0.07	0.26	-0.20*	0.03	0.56	-0.71	-2.06**	0.01	-5.89*
Belgium	-0.26	0.22	0.34	0.02	-0.30**	-0.34	-1.31***	-1.27	-1.13	-0.03***	-5.57*
Norway	-0.09	-0.00	0.25	0.13	-0.31*	-0.34	0.06	-1.11	-1.81**	-0.06*	-5.56*
Austria	0.02	-0.09	0.22	0.16	-0.25**	-0.50	0.77	-1.35	-2.16***	-0.05*	-5.24*
Czech Rep.	-0.19	-0.09	0.20	0.13	-0.21***	0.20	0.86	-1.77***	-3.31***	-0.08*	-4.75*
Estonia	0.07	-0.10	0.08	0.14	-0.19	-0.83	1.01	-0.24	-1.43	-0.06*	-5.45*
Germany	-0.10	-0.02	-0.24	0.41	-0.12	-0.40	0.09	1.33	-3.36***	-0.02***	-5.50*
Russia	0.11	0.10	-0.01	-0.21	-0.21***	-1.18***	-0.12	-0.34	-0.49	-0.00	-4.98*
US	0.01	-0.05	0.09	-0.06	-0.37*	0.37	0.72	-0.60	-0.67	0.01*	-4.73*

*** $p<.001$; ** $p<.01$; * $p<.05$
